# Effect of tailored use of tirofiban in patients with Non-ST-elevation acute coronary syndrome undergoing percutaneous coronary intervention: a randomized controlled trial

**DOI:** 10.1186/s12872-018-0938-6

**Published:** 2018-10-22

**Authors:** Wonjae Lee, Jung-Won Suh, Jin Joo Park, Chang-Hwan Yoon, Young-Seok Cho, Tae-Jin Youn, In-Ho Chae

**Affiliations:** 1 Division of Cardiology, Department of Internal Medicine, College of Medicine, Seoul National University, Seongnam-si, Gyeonggi-do Korea; 2 Cardiovascular Center, Seoul National University Bundang Hospital, Seongnam-si, Gyeonggi-do Korea

**Keywords:** Glycoprotein IIb/IIIa inhibitor, Tailored antiplatelet treatment, Periprocedural myonecrosis, High residual platelet activity

## Abstract

**Background:**

We conducted a randomized controlled trial to investigate whether an additional platelet inhibition with tirofiban would reduce the extent of myocardial damage and prevent periprocedural myonecrosis in patients with Non-ST-elevation acute coronary syndrome (NSTE-ACS) with a high residual platelet activity (HPR).

**Methods:**

Patients with an HPR, defined as P_2_Y_12_ reaction unit (PRU) > 230, were randomly assigned to group A (tirofiban treatment, *n* = 30) or C1 (*n* = 30) and patients without an HPR to C2 (*n* = 78). Periprocedural myocardial damage was assessed using the area under the curve (AUC) of serial cardiac enzyme levels from the time of the procedure to post-36 h. Periprocedural myonecrosis incidence was evaluated.

**Results:**

The troponin I AUC was not different between the groups (197.2 [41.5395.7], 37.9 [8.9313.9], 121.3 [43.7481.8] h∙ng/mL; *p* = 0.088). The results did not change when the baseline levels were adjusted (365.3 [279.5, 451.1], 293.0 [207.1, 379.0], and 298.0 [244.7, 351.3] h∙ng/mL; *p* = 0.487). The rate of periprocedural myonecrosis was also not different between the groups (53.0% vs. 50.0% vs. 33.3%, *p* = 0.092). The CK-MB isoenzyme analysis showed similar results. No difference in complications was noted.

**Conclusion:**

Additional tirofiban administration was not beneficial to patients with NSTE-ACS even with an HPR.

**Trial registration:**

Clinical trial no. NCT03114995, registered 11 April, 2017, retrospectively.

**Electronic supplementary material:**

The online version of this article (10.1186/s12872-018-0938-6) contains supplementary material, which is available to authorized users.

## Background

Dual antiplatelet therapy (DAPT) plays a fundamental role in patients undergoing percutaneous coronary intervention (PCI). A decreased response to clopidogrel is related to higher myocardial damage, thus leading to a worse outcome after PCI [1, 2]. Numerous studies have been conducted to improve the outcomes by intensifying the antiplatelet therapy with intravenous glycoprotein IIb/IIIa (GP IIb/IIIa) inhibitors and newer-generation P_2_Y_12_ inhibitors [3–6]. However, using these drugs in all patients undergoing PCI does not seem cost-effective and increases unwanted bleeding events [7, 8]. Therefore, identifying patients who have a poor response to their current DAPT and require an additional antiplatelet treatment is critical. Ultegra Rapid Platelet Function Analyzer (VerifyNow®) is a point-of-care assay tool that can easily assess platelet reactivity after the administration of clopidogrel and aspirin. Studies showed that platelet reactivity inhibition measured by this device can predict the prognosis of patients who undergo PCI [9]. Tirofiban, a GP IIb/IIIa inhibitor, was known to be beneficial for broader sets of patients with acute coronary syndrome (ACS); however, recent studies have shown a limited role of tirofiban except for high-risk patients with Non-ST-elevation ACS (NSTE-ACS) undergoing an early invasive strategy [10–12].

We hypothesized that patients with NSTE-ACS stabilized with standard medical treatment can benefit from adding tirofiban to DAPT when they undergo PCI if they have a high platelet reactivity (HPR) identified using VerifyNow®.

## Methods

### Study design

This was a prospective randomized clinical study conducted at Seoul National University Bundang Hospital from February 2012 to October 2015.

We consecutively enrolled patients who are already stabilized with standard medical treatment and diagnosed with NSTE-ACS. Patients had been loaded with aspirin and clopidogrel at least 6 h before the procedure. Patients were excluded if they were < 18 or ≥ 85 years old and had a contraindication for antiplatelet treatment, thrombocytopenia (platelet count < 100,000/μL), history of hemorrhagic stroke, history of ischemic stroke in the recent 2 years, or history of major surgery 6 months prior. All patients provided written informed consents, and the study was authorized by the local institutional review board. The full protocol of the present study has been registered at http://www.clinicaltrials.gov (clinical trial no. NCT03114995).

**Fig. 1:**
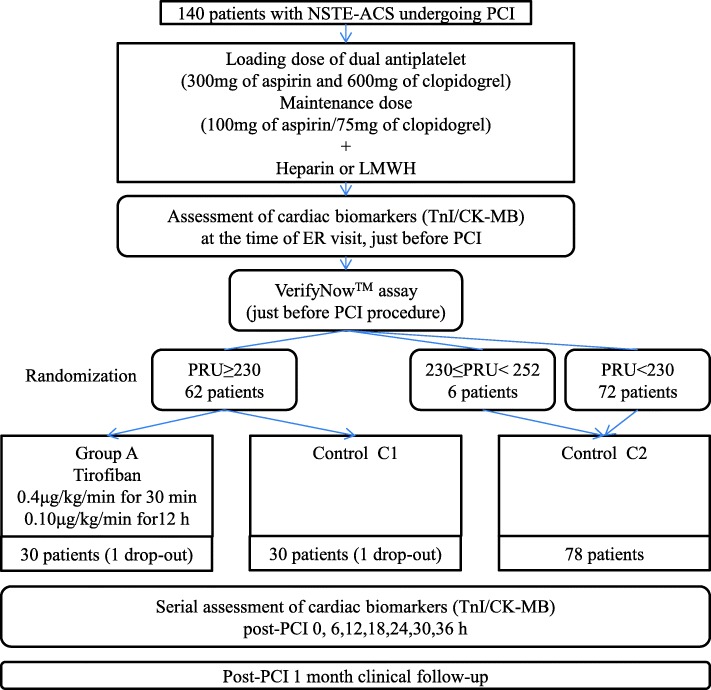
Study flow of the randomized controlled trial

Figure 1 summarizes the flow of this study. The standard loading doses were 300 mg of aspirin and 600 mg of clopidogrel. We administered a maintenance dose of aspirin 100 mg/d and clopidogrel 75 mg/d to all patients. The VerifyNow P_2_Y_12_ assay was used right before PCI at the catheterization laboratory. Based on previous study in our center, the sample size and cutoff value were determined [13].

When the P_2_Y_12_ reaction units (PRU) were reported, we designated patients with a cutoff value of ≥252 as the HPR group and randomized them into group A or control group C1. Computerized-random number table was used to generate the sequence, and patients were allocated to each group following simple randomization procedure. Randomization process were conducted by the research nurses right after PRU value were obtained. Patients without an HPR were allocated to control group C2. However, we adjusted the cutoff value of PRU to 230 after enrolling 42 patients (30 patients to control group C2), since there were fewer patients with HPR than the estimated number in the initial enrollment phase. Group A was treated with tirofiban (0.4 μg/kg/min continuous infusion for 30 min and then 0.10 μg/kg/min continuous infusion for 12 h) in addition to heparin (70 U/kg intravenous bolus infusion), while the control groups were administered only with heparin (140 U/kg intravenous bolus infusion). The level of cardiac biomarkers was measured right before the procedure and serially after the procedure at post-6, 12, 18, 24, 30, and 36 h. The cardiac biomarkers used in this study were cardiac troponin I (TnI: VITROS 5600 System, Ortho Clinical Diagnostics, Raritan, New Jersey, USA) and creatine kinase-MB isoenzyme (CK-MB, Dimension Vista 1500 system, Siemens Healthcare Diagnostics, Munich, Germany). All patients were followed up after 1 month to evaluate the clinical outcomes.

### Primary, secondary objectives, and safety results

The primary objective of this study was to compare the myocardial damage primarily related to the procedure between the groups. The damage was assessed using the area under the curve (AUC) of the serial cardiac biomarker levels from the time of the procedure to post-36 h. AUC was calculated using the trapezoidal method. The adjusted AUC was calculated to exclude the differences in the cardiac biomarker level owing to the index MI event between the groups. The secondary objective was to evaluate the prevalence of periprocedural myonecrosis. We referenced the 2012 Third Universal Definition of Myocardial Infarction to determine the events of periprocedural myonecrosis with TnI and CK-MB. When the cardiac biomarkers before the procedure were within the 99th percentile upper reference limit (URL), more than a 5-fold elevation in the URL within 12 h after PCI was defined as periprocedural myonecrosis. If the cardiac biomarker level was already above the 99th percentile URL before the procedure and the trend was stationary or decreasing, a ≥ 20% increase compared to the previous level was considered periprocedural myonecrosis. If the trend was still increasing, the levels at the post-6 h and 12-h were compared to determine periprocedural myonecrosis. The PCI-related findings, including the involved vessels, the number of stents used, and immediate post-PCI complications were analyzed. Major adverse cardiac events, including the composite of cardiac death, nonfatal spontaneous myocardial infarction, and urgent target vessel revascularization, were evaluated at 1-month follow-up visit. Other adverse events, including bleeding, were also assessed. The TIMI criteria were used to classify bleeding complications into minimal, minor, and major.

### Statistical analysis

We planned to recruit 140 patients, expecting 80 patients to have a PRU ≥252 with the assumption of a 4:3 ratio in the general population. The assumption including cutoff value of PRU was derived from previous studies conducted in our center with Korean population [13]. We used the SPSS, version 17.0 (IBM, New York, New York, USA) to perform the statistical analyses. Continuous variables were presented as means±SD or medians [interquartile ranges (IQR)], and categorical variables as crude numbers and percentages. The student’s t-test was used to compare continuous variables and the chi-square or Fisher’s exact test to compare the frequency with categorical variables for the baseline characteristics. To compare the AUCs between the groups, the Kruskal-Wallis test and the Mann-Whitney U test with Bonferroni method for post hoc comparison were conducted. The adjusted AUC was compared while controlling for the initial cardiac enzymes using the one-way analysis of covariance. The results were shown in mean and 95% confidence interval. The chi-square test was used to analyze the periprocedural myonecrosis. Two-sided *p* values < 0.05 were considered significant.

## Results

### Study population

**Fig. 2:**
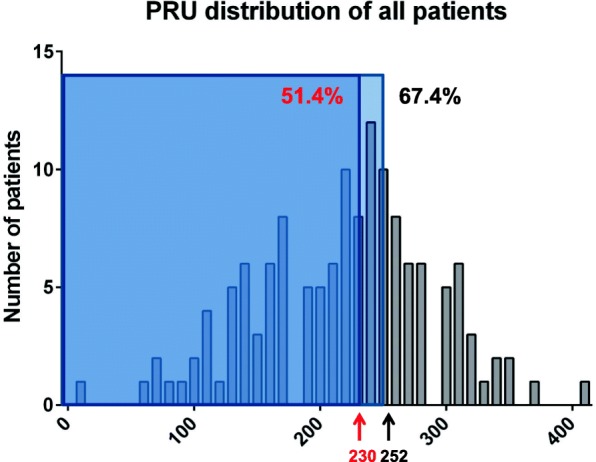
Histogram showing the distribution of P_2_Y_12_ reaction units of all patients. The cut-off values of 230 and 252 are indicated to show the proportion of patients within the ranges

In this study, 140 patients with NSTE-ACS undergoing PCI were enrolled during 44 months as planned. One patient in group A and 1 patient in control group C1 were dropped. Figure 2 shows the number of patients allocated to each group. Since there was a change in the cutoff value of PRU, 6 patients in the initial enrollment phase who had a PRU value between 230 and 252 were assigned to control group C2. In total, around half (48.6%) of the patients had a PRU value ≥230 as shown in Fig. 2.

### Baseline characteristics and angiographic and PCI findings

Tables 1 and 2 show the baseline characteristics and PCI and angiographic findings. There was no difference between group A and control group C1 in the baseline characteristics, except for the number of men in control group C1 (control group C1 had a higher number of men [83.3% vs 56.7%, *p* = 0.048]). In the angiographic findings, group A tended to have more patients with multi-vessel PCI that was not statistically significant (group A: 43.3%, control group C1: 30.0%, *p* = 0.421). Otherwise, no differences were observed. Between control groups C1 and C2, the hematocrit level was significantly lower in control group C1 than in control group C2 (41.3 ± 4.3% vs 43.5 ± 4.4%, *p* = 0.022). The low-density lipoprotein cholesterol level was also lower in control group C1. There was no difference in the angiographic findings. Group A and control group C2 had a difference in the baseline characteristics, such as in sex, age, and diabetes. Group A had more stents per target lesion than control group C2 (1.20 ± 0.41 vs 1.05 ± 0.23, *p* = 0.009).

**Table 1:** Baseline characteristics, laboratory findings, and P_2_Y_12_ reaction units

Variables	Group A	Control C1	*p* Value	Control C2	*p* Value	*p* Value
			(A vs C1)		(C1vs C2)	(A vs C2)
Demographic characteristics						
Men (%)	17 (56.7%)	25 (83.3%)	0.048	67 (85.9%)	0.973	0.002
Age (years)	70.0 ± 12.8	64.5 ± 12.0	0.091	62.9 ± 10.1	0.486	0.003
Body mass index (kg/m^2^)	25.1 ± 3.0	24.6 ± 2.0	0.474	25.2 ± 3.1	0.852	0.320
Diabetes	14 (46.7%)	8 (26.7%)	0.180	17 (21.8%)	0.777	0.020
Hypertension	19 (63.3%)	17 (56.7%)	0.792	35 (44.9%)	0.376	0.132
Medication at ER						
Aspirin loading	27 (90.0%)	22 (73.3%)	0.182	70 (89.7%)	0.064	1.000
Clopidogrel loading	27 (90.0%)	27 (90.0%)	1.000	76 (97.4%)	0.256	0.256
Beta blocker	26 (89.7%)	23 (76.7%)	0.326	71 (91.0%)	0.095	1.000
ACEI/ARB	23 (79.3%)	25 (83.3%)	0.950	63 (80.8%)	0.975	1.000
CCB	1 (3.4%)	1 (3.3%)	1.000	13 (16.7%)	0.126	0.139
Statin	28 (96.6%)	30 (100.0%)	0.986	77 (98.7%)	1.000	1.000
Major laboratory findings						
Hematocrit (%)	39.7 ± 5.8	41.3 ± 4.3	0.107	43.5 ± 4.4	0.022	0.000
Platelet (×10^3^/μl)	213.6 ± 49.0	226.1 ± 63.2	0.396	220.9 ± 47.0	0.639	0.483
Total cholesterol (mg/dl)	162.9 ± 35.6	176.1 ± 51.1	0.261	189.9 ± 38.5	0.132	0.002
Triglyceride (mg/dl)	129.6 ± 85.7	133.5 ± 92.6	0.867	133.1 ± 81.1	0.982	0.848
LDL cholesterol (mg/dl)	98.4 ± 40.3	100.2 ± 36.5	0.861	116.9 ± 33.5	0.029	0.022
Serum creatinine (mg/dl)	0.9 ± 0.6	0.9 ± 0.2	0.819	0.8 ± 0.2	0.262	0.331
Ejection fraction (%)	57.1 ± 9.1	59.8 ± 6.2	0.201	59.6 ± 6.5	0.852	0.134
P2Y_12_ reaction units	277.2 ± 39.2	281.4 ± 38.4	0.672	171.1 ± 51.6	0.000	0.000

*ER* emergency room, *ACEI* angiotensin-converting enzyme inhibitor, *ARB* angiotensin-receptor blocker, *CCB* calcium channel blocker, *LDL* low-density lipoprotein

**Table 2:** Percutaneous coronary intervention procedure and angiographic findings

Variables	Group A	Control C1	*p* Value	Control C2	*p* Value	*p* Value
			(A vs C1)		(C1vs C2)	(A vs C2)
Lesion characteristics						
Multivessel PCI	13 (43.3%)	9 (30.0%)	0.421	25 (32.1%)	1.000	0.381
Target vessel						
Left main disease	0 (0.0%)	1 (3.3%)	1.000	5 (6.4%)	0.875	0.378
Left anterior descending artery	18 (60.0%)	19 (63.3%)	1.000	47 (60.3%)	0.941	1.000
Left circumflex artery	11 (36.7%)	11 (36.7%)	1.000	31 (49.7%)	0.941	0.941
Right coronary artery	11 (36.7%)	11 (36.7%)	1.000	20 (25.6%)	0.370	0.370
IVUS guidance	9 (31.0%)	4 (13.3%)	0.184	21 (26.9%)	0.213	0.858
Type B2/C lesion	36 (73.5%)	35 (79.5%)	0.657	90 (81.8%)	0.922	0.323
Restenotic lesion	2 (2.3%)	1 (4.3%)	1.000	0 (0.0%)	0.634	0.161
Angiographic thrombus	13 (43.3%)	11 (36.7%)	0.792	30 (38.5%)	1.000	0.807
Procedure characteristics						
Stents per target lesion (n)	1.20 ± 0.41	1.09 ± 0.29	0.124	1.05 ± 0.23	0.460	0.009
Stent type						
Drug eluting balloon	1	0		0		
Bare metal stent	0	1		0		
Drug-eluting stent	53	47		117		
Stent size	2.70 ± 0.47	2.79 ± 0.46	0.651	2.84 ± 0.45	0.409	0.737
Stent length	23.7 ± 8.7	21.8 ± 5.5	0.548	23.0 ± 8.7	0.613	0.838
Duration of loading time to the procedure	34.0 ± 18.6	29.9 ± 18.3	0.480	29.4 ± 16.1	0.917	0.270
Emergency PCI	1	1		0		

*PCI* percutaneous coronary intervention, *IVUS* intravascular ultrasound

### Comparison of the primary and secondary outcomes

**Fig. 3:**
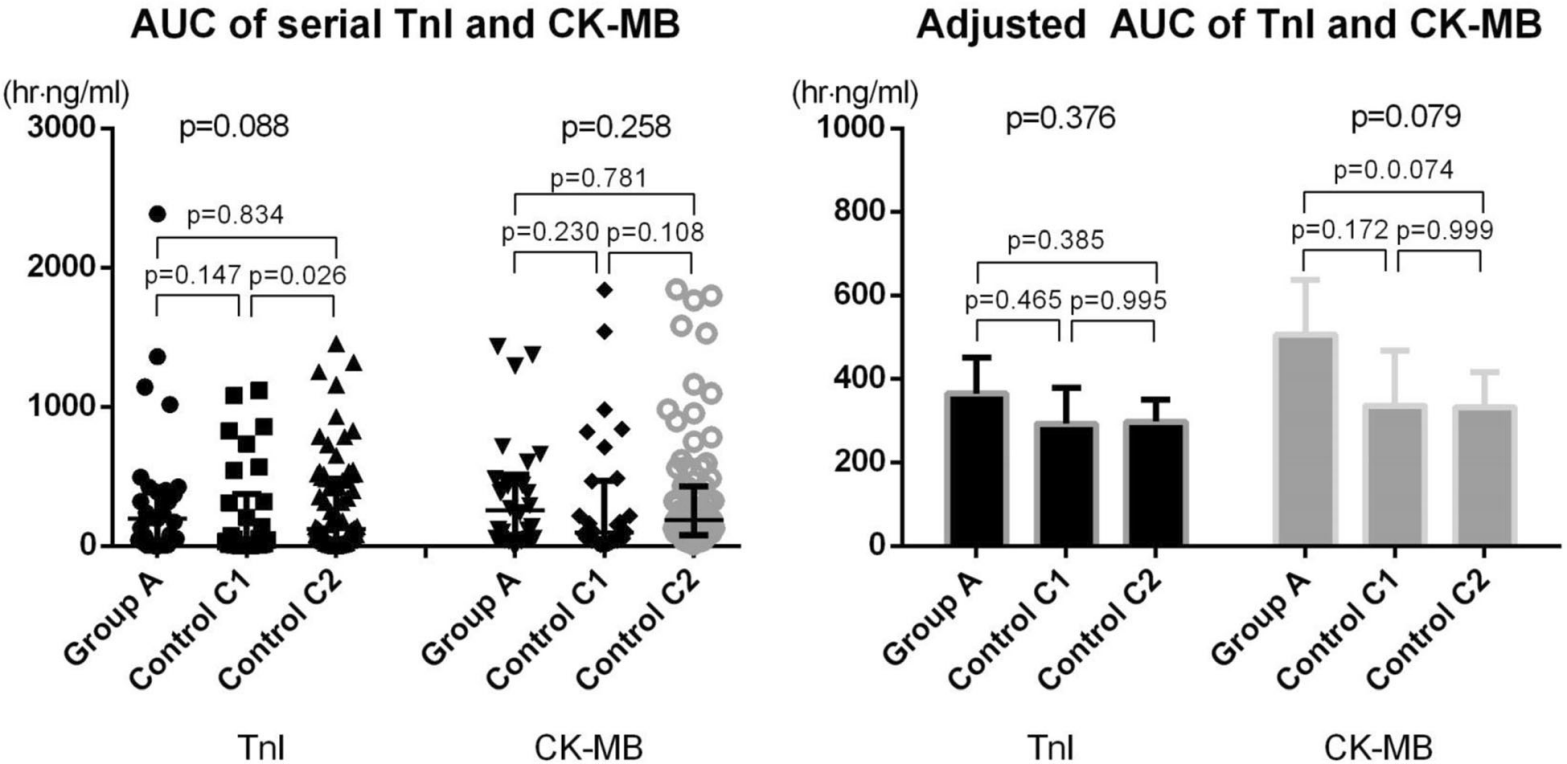
Comparison of area under curves of serial troponin I and creatine kinase-MB measurements between groups

The primary endpoint, AUCs of TnI and CK-MB, which represent the extent of periprocedural myocardial damage, was not different among the three groups (TnI, h∙ng/mL: 197.2 [41.5395.7] vs 37.9 [8.9313,9] vs 121.3 [43.7481.8], *p* = 0.088; CK-MB, h∙ng/mL: 252.5 [48.0,470.1] vs 92.7 [39.1402.1] vs 185.6 [79.7425.3], *p* = 0.258; Fig. 3). The post-hoc comparison between group A and control group C1 showed no difference (TnI: *p* = 0.147; CK-MB: *p* = 0.230). The AUCs were not different group A and C2 (TnI: *p* = 0.834; CK-MB: *p* = 0.781). The AUCs of TnI and CK-MB adjusted by the initial level of biomarkers are shown in Fig. 3. The adjusted AUCs (h∙ng/mL) of TnI were 365.3 [279.5, 451.1], 293.0 [207.1, 379.0], and 298.0 [244.7, 351.3]. The adjusted AUCs (h∙ng/mL) of CK-MB were 505.9 [373.7, 638.2], 336.2 [204.1, 468.3], and 333.2 [251.2, 415.2]. The adjusted AUCs confirmed that no difference exists in periprocedural myocardial damage between the groups (TnI: group A vs control group C1, *p* = 0.465; group A vs control group C2, *p* = 0.385; CK-MB: group A vs control group C1, *p* = 0.172; group A vs control group C2, *p* = 0.074) in the post-hoc comparison.

**Fig. 4:**
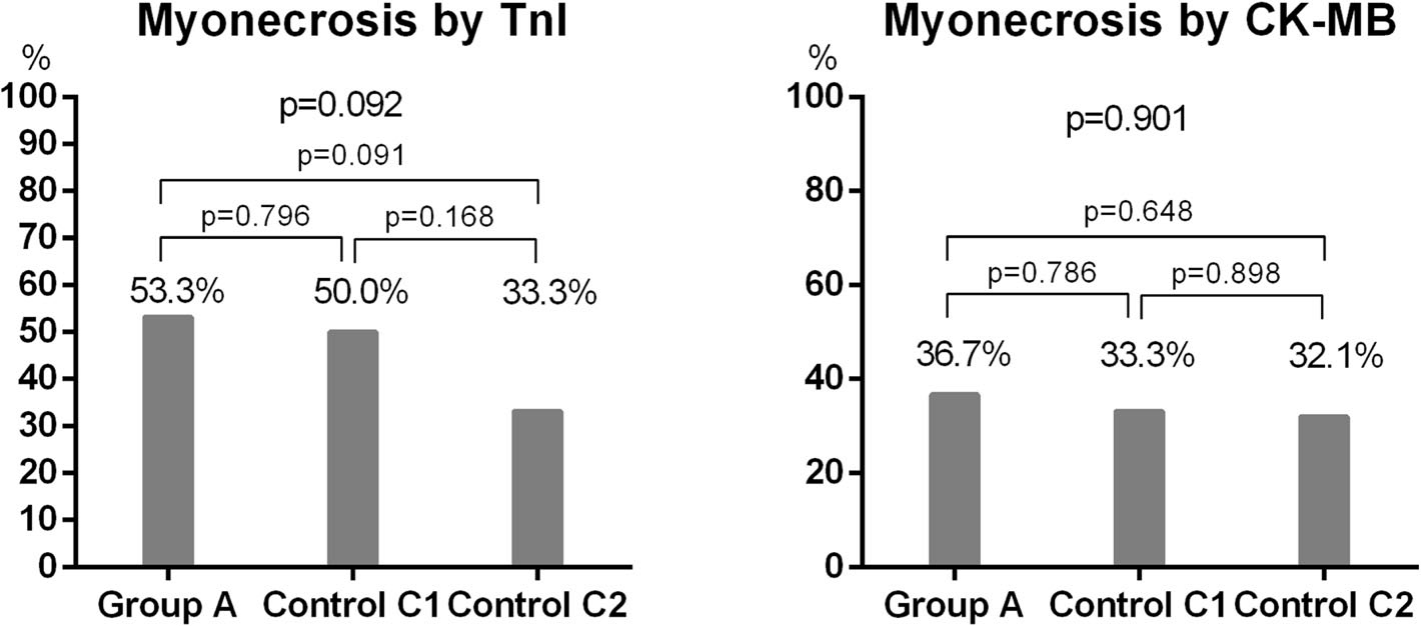
Incidence of PMI by Troponin I and creatinine kinase-MB

The rates of periprocedural myonecrosis, the secondary endpoint, are shown in Fig. 4. The rates of periprocedural myonecrosis by TnI were 53.3% in group A, 50.0% in control group C1, and 33.3% in control group C2 (*p* = 0.092). The rates between the groups were not different (group A vs control group C1, *p* = 0.796; group A vs control group C2, *p* = 0.091). The rates of periprocedural myonecrosis by CK-MB in group A, control group C1, and control group C2 were 36.7%, 33.3%, and 32.1%, respectively (*p* = 0.901). The comparison between each group also showed no intergroup difference in the incidence of periprocedural myonecrosis (group A vs control group C1, *p* = 0.786; group A vs control group C2, *p* = 0.648).

### Analysis of adverse outcomes

There were 2 major adverse cardiac events at 1-month follow-up only in group A. One patient had a massive hematochezia two week after the PCI due to early rectal cancer and died. The other patient died 3 days after the PCI, presumably due to a subacute stent thrombosis. Although the difference in the event rate was not statistically significant between the 3 groups, the mortality cases in group A should be noted. Except for the abovementioned case of hematochezia, no major bleeding was observed in all groups. The event rate of minor to minimal bleeding, such as small hematomas, in group A was the highest with 13.3% among the groups (control group C1: 3.3%; control group C2: 10.3%); however, the differences were not statistically significant.

### Subgroup analysis with PRU ≥ 252

**Fig. 5:**
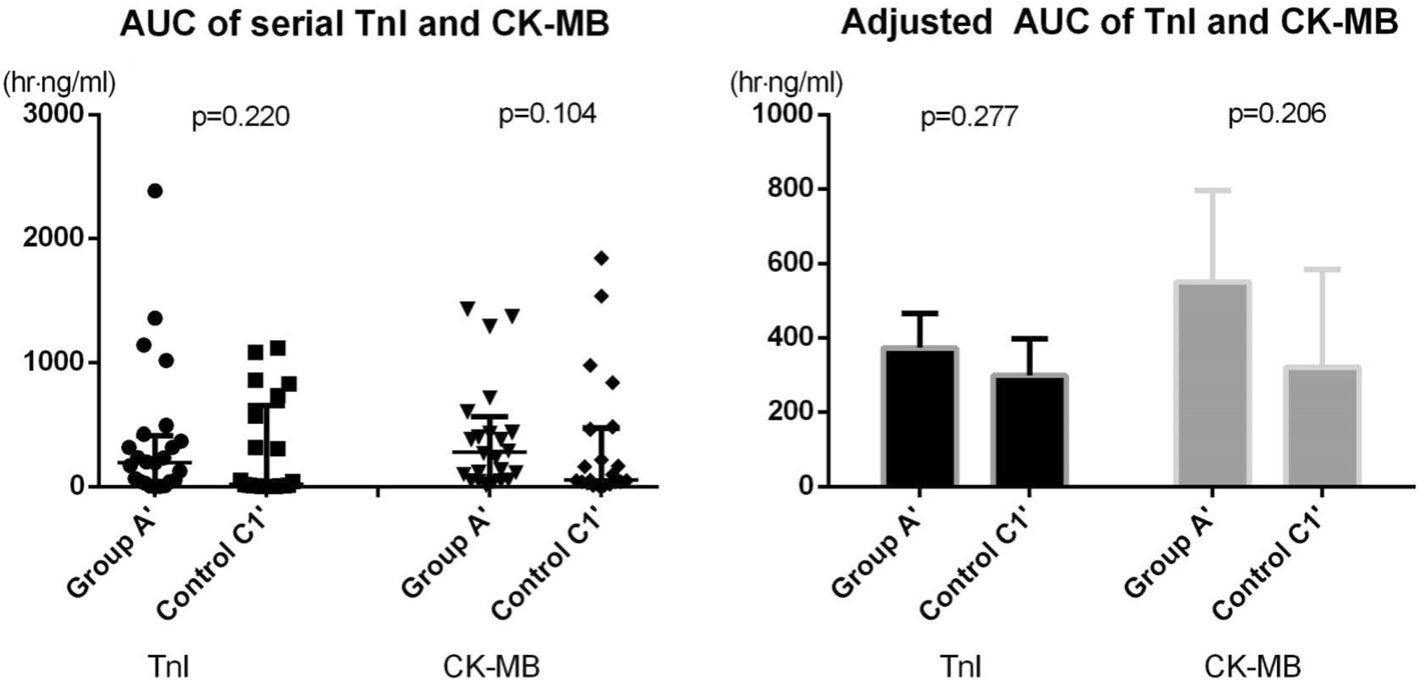
Subgroup analysis in patients with platelet reactivity unit≥252 comparing area under curves of serial troponin I and creatine kinase-MB measurements between groups

Since the PRU cutoff value 230 could not show any difference in the primary endpoints, we did a subgroup analysis using a PRU 252, a cutoff value initially proposed. Twenty-four patients in group A (group A’) and 21 patients in control group C1 (control C1’) were analyzed. The subgroup analysis did not show any difference between the 2 groups (Fig. 5); the AUCs by TnI were 197.2 [43.2, 383.3] and 17.6 [8.9, 566.5] h∙ng/mL (*p* = 0.220), and that by CK-MB were 278.5 [86.6, 477.1] and [53.6 [38.7, 464.2] h∙ng/mL (*p* = 0.104), respectively. The adjusted AUCs were also similar between group A’ and control C1’ (TnI, h∙ng/mL: 373.1 [279.7, 466.4] vs. 298.5 [198.8, 398.3], *p* = 0.277; CK-MB, h∙ng/mL: 550.1 [304.0, 796.2] vs. 320.4 [57.3, 583.5], *p* = 0.206).

## Discussion

We assessed the benefit of the tailored antiplatelet therapy with GP IIb/IIIa inhibitor tirofiban in patients with NSTE-ACS undergoing PCI who were stabilized with standard medical treatment. Tirofiban did not either reduce the extent of myocardial damage or the incidence of periprocedural myonecrosis in patients who had an HPR defined by a PRU ≥ 230. Although tirofiban tended to increase bleeding events, they were mostly minor, which were not significant and similar to the control groups. A noteworthy finding was that approximately half of the patients enrolled did not achieve an adequate response to clopidogrel before PCI even though clopidogrel was loaded 30.4 ± 17.1 h before PCI on average.

### Conflicting results of tailored antiplatelet therapies

A meta-analysis by Daniel et al. showed that a high clopidogrel platelet reactivity measured by an ADP-specific platelet function assay is a strong predictor of major adverse cardiac events in patients after PCI [1]. It was reported that platelet reactivity assessed using the point-of-care assay VerifyNow had a prognostic significance on thrombotic events after drug-eluting stent implantation [9]. Boosted by these findings, series of studies have been conducted to assess the efficacy of a tailored antiplatelet treatment in patients undergoing PCI. A landmark study conducted by Bonello et al. demonstrated that the rate of stent thrombosis and major adverse cardiac events was significantly lower in patients undergoing PCI when a tailored clopidogrel loading dose was given [14]. Patients were administered up to 3 additional loading doses of clopidogrel to achieve an adequate inhibition assessed by the vasodilator-stimulated phosphoprotein index. Valgimigli et al. reported that tirofiban lowered the incidence of MI after elective coronary intervention when administered to low-risk patients who had a poor responsiveness to standard oral platelet inhibitors [15]. A recent observational study conducted by Dridi et al. also showed that patients exhibiting an HPR benefited from the tailored antiplatelet therapy with either a double-dose clopidogrel or the newer P_2_Y_12_-inhibitors [16]. However, large clinical trials reported different results on tailored antiplatelet therapies. The GRAVITAS randomized trial, which enrolled 2214 patients with stable angina and NSTE-ACS, concluded that the use of a high-dose clopidogrel in non-responders did not reduce the incidence of ischemic events [17]. In the ARCTIC trial that studied 2440 patients scheduled for an elective PCI, the investigators administered an additional dose of clopidogrel, prasugrel, or aspirin with GP IIb/IIIa inhibitors to the monitored group if the patients had an HPR. The study did not show significant improvements in the clinical outcomes in the monitored group compared to the conventional treatment group without monitoring [18]. In line with these trials, we previously reported in the DM-Verify Now trial that a tailored antiplatelet therapy could not reduce periprocedural myonecrosis in patients with diabetes mellitus [19]. Our study results are consistent with those of previous large clinical trials, which could not demonstrate an improvement in the clinical outcomes of guided antiplatelet therapies with platelet function tests. To date, the current guidelines do not support the routine use of platelet function tests [20]. There were few studies conducted with newer P2Y12 agent for tailored antiplatelet treatment. Aradi et al. evaluated the impact of prasugrel and high-dose clopidogrel for HPR patient with ACS [21]. Switching to prasugrel resulted in better clinical outcome. ANTARCTIC trial is a large scale randomized controlled trial which compared conventional treatment and tailored antiplatelet treatment using prasugrel [22]. In the monitoring group, patient Patients in monitoring groups were tested with VerifyNow assay 14 days after initiation of prasugrel, and the dose of prasugrel of patients with platelet reactivity below 208 were increased to 10 mg. However, tailored antiplatelet treatment did not provide improved clinical outcomes.

It is possible that conflicting results may have resulted from different study protocols such as modality for measuring antiplatelet reactivity, cutoff values, and timing of measurement. A meta-analysis by Aradi et al. revealed that there were large inter-study and intra-assay heterogeneity in the prevalence of HPR that resulted in a range of 6% to 80%, driven by the differences in the test methods and cutoff values [1]. In fact, several representative trials such as GRAVITAS, TRIGGER-PCI, and ARTIC which used VerifyNow all failed to show improvement in clinical outcomes, whereas other studies which used VASP assay, LTA, or MEA demonstrated the benefit of tailored antiplatelet therapy [23]. Cutoff values for HPR of VerifyNow were mostly between 230 and 240, but recent studies such as ANTARTIC trial used PRU of 208 to define HPR [2, 18, 22]. Timing of measurement also varied. Many studies tested platelet reactivity at least 12 h after loading of clopidogrel, but some studies were as early as 6 h [14, 17, 24]. In GRAVITAS trial, on-treatment reactivity decreased significantly over the first 30 days, and the extent was different between standard dose and high dose clopidogrel while PRU in 48 h were similar [17]. Given that platelet reactivity is enhanced in patients with ACS at early phase, single measurement may not be sufficient to reflect patient’s risk especially in ACS [25, 26].

### Potential role of tirofiban in patients with an HPR

Earlier studies demonstrated that GP IIb/IIIa inhibitors reduce the mortality and other major adverse cardiac events in patients with ACS or undergoing PCI [27, 28]. Desai et al. pointed out that numerous early studies on GP IIb/IIIa inhibitors were conducted without a concomitant antiplatelet therapy with a thienopyridine, a prior standard treatment [12]. This implies that there may be an additional role of GP IIb/IIIa inhibitors for patients when the pretreatment with clopidogrel does not achieve an adequate platelet inhibition. Moreover, benefits could be more evident for patients with higher risks, such as elevated cardiac biomarker levels. However, our findings suggest that the GP IIb/IIIa inhibitor tirofiban does not provide an additional protection to the myocardium regardless of the platelet reactivity after the treatment with aspirin and clopidogrel. It is noteworthy that the subgroup analysis of the patients who had an elevated TnI before PCI showed no tirofiban benefits. One explanation is that our patients were already stabilized with conventional or low-molecular-weight heparin before PCI, thus reducing the thrombotic complications during PCI. In the ISAR-REACT 2 trial that showed the efficacy of abciximab in patients with NSTE-ACS, especially when the TnI level was elevated, clopidogrel loading was performed at least 2 h with an average of 6 h before PCI; in our trial, it was performed 31.5 h on average, which may have given enough time to stabilize patients with the full effects of heparinization.

### Limitations

The limitation of our study is that we adjusted the cutoff value early in the trial. As planned, 140 patients in total were enrolled in this study. However, we could not gather enough number of patients who have an HPR to clopidogrel for the study groups, since the distribution of the PRU value among the patients was not similar to a previous study conducted in our center. Park et al. suggested that platelet reactivity < 275 PRU is sufficient to achieve lower risks of cardiac death, MI, and stent thrombosis in Koreans. However, they also validated that platelet reactivity between PRU 230 to 240 is an important risk factor for primary outcome in multivariate analysis similar to previous studies with Caucasians [9, 24, 29]. Thus, we also applied a cutoff value of PRU 230, but the low cutoff value may have limited the power to discern the high-risk group that has resistance to clopidogrel, masking the additive effect of tirofiban. To mitigate the issue, we conducted subgroup analysis using cutoff value of 252 for comparison of group A and C1, and the results was not different. In addition, since 6 patients with 230 ≤ PRU < 252 who might have been randomized into Group A or C1 were included in group C2, we performed same analysis after excluding them to confirm there is no difference in result due to change of the cutoff value (Additional file 1: Figure S1 and Additional file 2: Figure S2). There were some differences in baseline characteristics that may have limited the positive results in the tirofiban group though statistically insignificant. The patients in the tirofiban group tended to have more multivessel PCIs and the higher initial value of TnI and CK-MB. This may have resulted from the relatively small number of patients in the study group which could lead to an unsatisfactory randomization. However, we conducted an adjusted AUC analysis that can partly mitigate these issues, and the results were not different. Finally, recent guidelines suggest that prasugrel or ticagrelor should be used in high-risk patients undergoing PCI to overcome antiplatelet resistance [11]. Thus, it may be simpler to use prasugrel or ticagrelor instead of clopidogrel with PRU guidance. However, experts’ consensus is that Asian population has different risk profile on thrombophilia and bleeding compared to Caucasian [30]. The PRASFIT-ACS showed the lower dose of Prasugrel has similar efficacy with lower risk of bleeding in Japanese patients with ACS [31]. Above all, newer-generation P_2_Y_12_ inhibitors were not available in Korea when the study was designed. Finally, long recruitment period is another limitation of this study.

## Conclusion

We showed that tirofiban infusion in patients with NSTE-ACS who are poor responders to clopidogrel could not decrease the extent of periprocedural myocardial damage and the rate of periprocedural myonecrosis. Our study suggests that further trials are needed to clarify further the benefit of tailored antiplatelet therapies in patients undergoing PCI.

## Additional files

Additional file 1: **Figure S1.** Comparison of area under curves of serial troponin I and creatine kinase-MB measurements between groups (6 patients with PRU higher than 252 in control C2 excluded). (PDF 123 kb)

Additional file 2: **Figure S2.** Incidence of PMI by Troponin I and creatinine kinase-MB (6 patients with PRU higher than 252 in control C2 excluded). (PDF 76 kb)

Additional file 3: Raw data. (XLSX 343 kb)

## Abbreviations

ACS: Acute coronary syndrome
AUC: Area under curve
CK-MB: Creatine kinase-MB isoenzyme
DAPT: Dual antiplatelet therapy
GP IIb/IIIa: Glycoprotein IIb/IIIa
HPR: High platelet reactivity
IQR: Interquartile ranges
NSTE-ACS: Non-ST-elevation ACS
PCI: Percutaneous coronary intervention
PRU: P_2_Y_12_ reaction units
TnI: Troponin I
URL: Upper reference limit
