# Supplementary figures and images for: Effect of tailored use of tirofiban in patients with Non-ST-elevation acute coronary syndrome undergoing percutaneous coronary intervention: a randomized controlled trial
Source: BMC Cardiovasc Disord. 2018 Oct 22;18:201. doi: 10.1186/s12872-018-0938-6 (PMC6198526; doi:10.1186/s12872-018-0938-6)

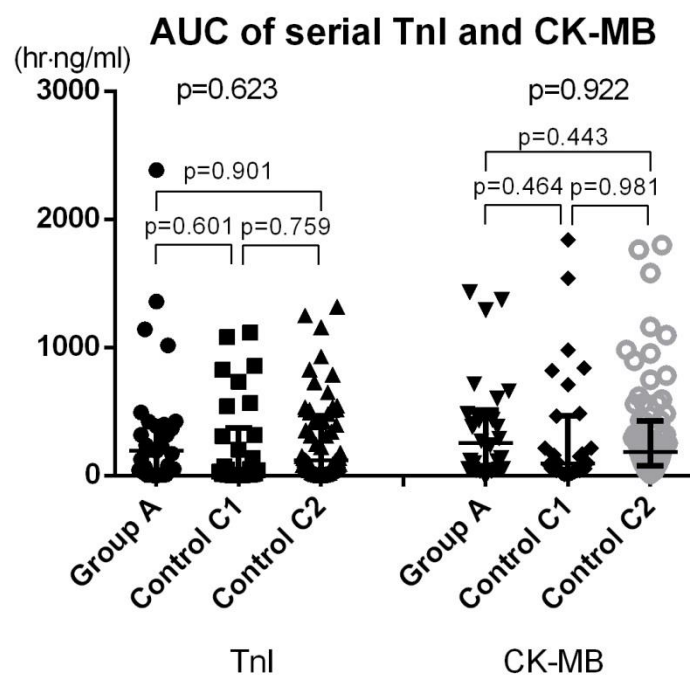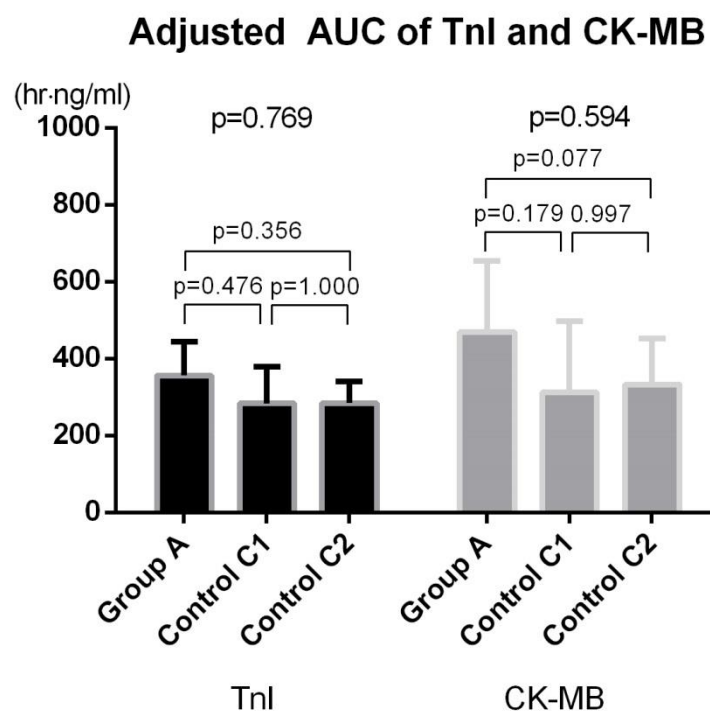

Supplement: Supplementary file 1 — Figure S1. Comparison of area under curves of serial troponin I and creatine kinase-MB measurements between groups (6 patients with PRU higher than 252 in control C2 excluded). (PDF 123 kb) [file 12872_2018_938_MOESM1_ESM.pdf]

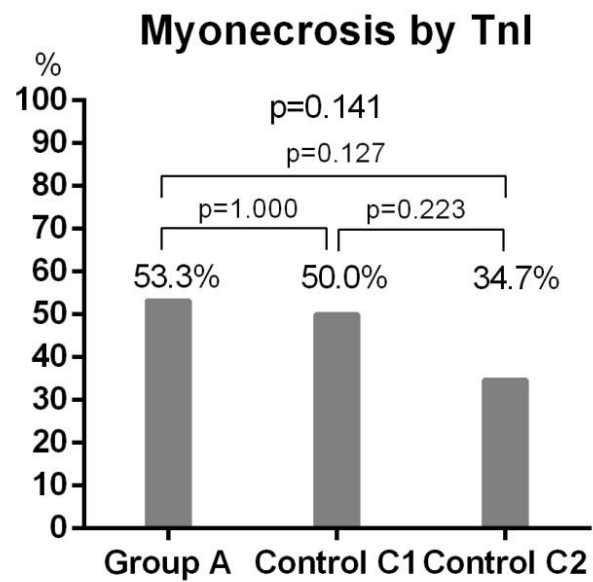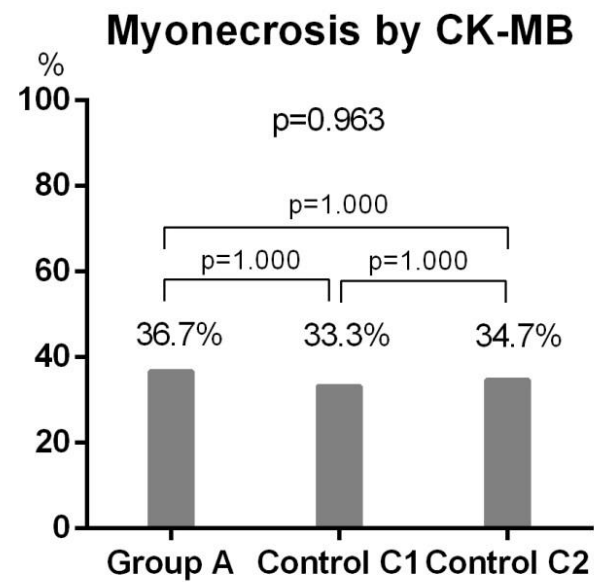

Supplement: Supplementary file 2 — Figure S2. Incidence of PMI by Troponin I and creatinine kinase-MB (6 patients with PRU higher than 252 in control C2 excluded). (PDF 76 kb) [file 12872_2018_938_MOESM2_ESM.pdf]
